# Educational adaptation to clinical training during the COVID-19 pandemic: a process analysis

**DOI:** 10.1186/s12909-022-03237-6

**Published:** 2022-03-23

**Authors:** Kristina Dzara, Martin Pusic, Narath Carlile, Edward Krupat, Erik K. Alexander

**Affiliations:** 1 Harvard Medical School, Boston, MA USA; 2 Brigham and Women’s Hospital, Brigham Education Institute, 75 Francis Street, TH-1-127, Boston, MA 02115 USA

**Keywords:** COVID-19, Medical Education, Virtual Learning, Change Management, Crisis Response

## Abstract

**Background:**

The COVID-19 pandemic is unprecedented in terms of the extent and rapidity of the disruption forced upon formal clinical education, most notably the extensive transition of clinical skills learning to interactive video-based clinical education.

**Methods:**

In a phenomenologic study, we used thematic analysis to explore the COVID-19 disruption to clinical training and understand processes relating to adaptation in a large academic medical center. We conducted semi-structured interviews with 14 clinical teachers and 16 trainees representing all levels of clinical learning. Interviews occurred within the initial three months of the crisis, and data were analyzed following a thematic analysis coding process.

**Results:**

We constructed eight themes synthesizing our participants’ perceptions of the immediate unanticipated disruption, noting in the process their alignment with a change management framework. These included: urgency in adapting, with an obvious imperative for change; overcoming inconsistent involvement and support through the formation of self-organized frontline coalitions; attempts to develop strategy and vision via initially reactive but eventually consistent communication; empowering a volunteer army through co-creation and a flattened hierarchy; and efforts to sustain improvement and positive momentum with celebration of trial, error, and growth. The majority of participants found positive outcomes resulting from the tumultuous change process. Moreover, they were now more readily accepting of change, and tolerant of the ambiguous and iterative nature inherent in the education change process. Many anticipated that some innovation would, or would at least deserve to, continue post- crisis.

**Conclusions:**

The COVID-19 pandemic afforded an opportunity to study the content and process of change during an active crisis. In this case of clinical education, our findings provide insight into the ways an academic medical system adapts to unanticipated circumstances. We found alignment with broader organizational change management models and that, compared with crisis management models (and their shorter term focus on resolving such crises), stakeholders self-organized in a reliable manner that carries the potential advantage of preserving such beneficial change.

**Supplementary Information:**

The online version contains supplementary material available at 10.1186/s12909-022-03237-6.

## Background

Medical education programs frequently undergo significant changes in their pedagogical approaches. Such changes are typically incremental, evolving slowly over time, and are guided by systematic planning by leadership [1–3]. A large number of barriers have been described for curricular and program changes, making the process often difficult and slow [4, 5].

The SARS CoV2 (COVID-19) pandemic forced immediate *unanticipated* change upon undergraduate (UME) and graduate (GME) medical education organizations, most notably in the capacity of trainees to continue traditional in-person learning.[6–14]. Within two weeks, the platforms and processes commonly used for nearly all medical learning were transformed [7, 8, 15]. The majority of non-direct clinical education was transitioned to interactive video-based clinical education, even though relatively few teachers or learners had significant experience with these technologies [6, 9–11, 16]. Few teachers or learners had ever experienced such a rapid disruptive change, with adaptation required at every level [6–8, 10, 11, 17].

Our motivation was to examine the phenomenon of sudden change in learning modality as it applies to clinical education at a large medical school. While a growing literature contains descriptions of COVID-19 pandemic effects on well-being and interpersonal engagement for clinicians and trainees [18–21], and of change and adaptation to the crisis in medical education [22–26], we sought to use the pandemic disruption as a lens on change management and crisis response. Specifically, we hoped to better understand the generalizable processes that result from a suddenly imposed and unexpected change upon the delivery of clinical skills education. Findings from this study could be applicable to both future imposed disruptions and to more intentionally planned instructional change.

## Methods

We investigated a phenomenon, the instructional modality shift in clinical education at Harvard Medical School brought on by the COVID-19 pandemic. Our data consisted of interviews with learners and teachers involved in clinical skills education. We used largely open-ended questions designed to elicit both positive and negative features of the then recent change. We designed the questions to throw light on our participants’ perspectives on change; however we did not start out with a specific research question nor impose a theoretical perspective on the data, as is appropriate for an inductive thematic analysis. After familiarizing ourselves with the data, we first generated codes that were subsequently integrated into themes [27, 28]. These initial themes were noted by the researchers to cohere when considered mainly at the *organizational* level and much less at the individual or local team levels. At that point, the investigators analyzed the data with a more specific perspective: that the modality shift could be broadly considered within an organizational change framework. Shifting to deductive theme development, we re-considered our codes and initial themes. As we describe below, we found considerable alignment with the Kotter framework for change management, though with important variations.

The research project was deemed exempt by the Mass General Brigham Institutional Review Board.

### Setting and participants

Our academic healthcare system converted all medical education efforts that did not involve physical provision of direct patient care (non-direct care) to the Zoom platform for interactive video-based clinical education within days of March 16^th^, 2020 as the pandemic worsened, inclusive of both graduate (GME) and undergraduate (UME) medical education programs. GME training encompasses a much larger portion of learning through direct care interaction than is the case for UME education. We use the term ‘interactive video-based clinical education’ to encompass the non-direct care teaching and learning process which occurred using Zoom as the digital medium. We focused our data collection exclusively on clinical skills learning across the continuum.

We collected data from medical teachers and learners actively involved in ongoing clinical education at our academic medical center who could provide insight both into the change in educational process and content as well as insight regarding change beneficiaries. Interviewees were purposefully recruited from teams and activities that represented the full spectrum of clinical training (excluding fellowship) including UME physical exam courses, UME core clinical clerkships, and GME general medical inpatient block rotations. The senior author (EKA) sent an email soliciting participation to two groups at our institution: 1) clinical students enrolled in their core clerkships, as well as all pre-clinical students enrolled in their clinical skills learning and 2) the faculty and resident trainees engaged with the inpatient teams of these students. While participation was voluntary, more than 70% agreed to participate. From the volunteers, we interviewed approximately equal numbers of teachers and learners, and stopped collecting data once interviews no longer lead to new changes in our coding or theme generation. We chose a particular instructional thread, “clinical education” – defined as learning to diagnose and manage patients – that would allow us to inspect change along the main continuum of learning at an academic health center, at both the UME and GME levels. Specifically, participants were drawn from the required doctoring course given in the preclinical years (2 learners and 3 instructors); the core clinical clerkships (10 learners and 8 instructors); and from the first years of two residency programs (Medicine and Pediatrics) (4 resident learners and 3 attending physician educators). Data collection and early analysis occurred concurrently; interviewers considered emerging consensus in their recruitment to ensure experiences could be adequately explored among target groups. We did not recruit senior administrators (e.g. the Deans), preferring to keep the focus on frontline clinical education, as opposed to the program as a whole.

### Interview guide

As we could identify no existing data collection tool or interview guide relating to the transition of clinical skills learning to interactive video-based clinical education during COVID-19, the research team collaboratively developed a semi-structured interview guide. The guide was refined prior to use following input from all team members, who collectively have experience in clinical medicine and educational research. The guide included 7 open ended questions eliciting positive and negative perceptions of the disruption, descriptions of changes to the nature and quality of clinical education, and expectations for ongoing adaptations during time of crisis (Additional File 1). While we would later come to use an organizational change framework as a sensitizing concept, this was not done tacitly at the time of initial interview guide development, as we initiated the study with few preconceived notions about what we might find due to the novel pandemic. Interviewers modified their process after defined stages of coding as described below [1, 2].

### Data collection

Recruitment for this study began on April 14th, 2020, with all interviews conducted from April 21st through May 16th, 2020. Thus, participant perspectives summarize experiences for approximately eight weeks through the time of greatest transition and adaptation. Interviews of approximately 30 min were conducted one on one via the Zoom[29] platform by team members (E.K.A., N.C.). Audio files were recorded and transcribed via the Zoom “auto-transcribe” feature. The interviewer then read, corrected, and finalized an accurate, de-identified transcript for study analysis.

### Data analysis

We followed an iterative thematic analysis coding process informed by Braun & Clarke[27] and Kiger and Varpio [28]. Initially, the first two transcripts– one from a clinical teacher and one from a clinical clerkship learner – were reviewed and open coded by each member of the research team. Coders initially focused on what change occurred and how, whether participants perceived that changes were successful, and lessons learned in the transition to the new learning environment. The first author then combined all codes into a preliminary codebook, which was reviewed and refined by the research team. The preliminary codebook was then applied again to the first two transcripts by all coders and no new codes were identified. The entire research team then met to perform a first iteration of mapping codes to early emerging themes. From this discussion, the first author created a coding template and revised codebook, which was reviewed and finalized by all team members. It was at this point that the investigators noted and incorporated the alignment with the change management framework as described below. We then reviewed the interview template to determine whether its structure was appropriate to the early emerging themes. Only small changes, mainly in emphasis, were made before the further interviews were carried out.

Aided by the coding template, each team member subsequently coded an additional five or six transcripts, stratified so as to include at least one medical student, resident, and clinician-educator. The codebooks were then combined by the first author, with codes grouped by constructed themes. The team reviewed codes and discussed how they mapped to the themes. Small, iterative changes to wording, order, and code structure were made, and codes which did not summarize data were removed. For each theme, code summaries were developed, reviewed, and compared to raw quotes. The team confirmed final themes based upon actively constructed patterns which aligned with an organizational change theory [27, 28].

Throughout our analysis, we considered how our roles might uniquely influence our findings. K.D. and E.K. are PhD educators and researchers who do not practice clinically. M.P. is an MD/PhD educator, researcher, and clinician who practiced clinically at the time of the study but did not teach or supervise any study participants. N.C. and E.K.A. are MD educators and clinicians who interacted with some participants in administrative, educational, or clinical settings, sometimes through direct supervision. All clinicians continued to practice clinically during the COVID-19 changes and adapted their practices to the disruption which may have influenced coding decisions. However, data coding efforts involved all team members to ensure that diverse perspectives were considered when interpreting data. Themes were reviewed and finalized by all team members, and those who taught or worked clinically confirmed credibility of the data.

## Results

The final products of our thematic analysis, informed by consideration of change management frameworks in general and the Kotter framework in particular, were eight key themes illuminating our participants’ perceptions of the immediate unanticipated disruption inherent in this transition to interactive video-based clinical education, highlighting the need for adaptability by both teachers and learners (Table 1).

**Table 1:** Summary of Kotter and Changes During COVID-19

*Classic Kotter*	*Feature*	*Change During COVID-19*	*Implication*
Establish a Sense of Urgency	● Consider potential future scenarios and untapped opportunities	● Urgency palpable	● Imperative for change was obvious
		● Level of clinical and personal danger prevalent	
	● Make the need for change clearly known	● Imperiled educational goals and metrics	
Form a Powerful Guiding Coalition	● Assemble a strong group of individuals	● Coalition emerges through differential engagement of participants	● Self-organized frontline coalition formed with technology adept teachers and learners in the lead efforts
	● Ensure the coalition will work well as a team towards the shared goal		
		● Co-creation prevalent	
		● Facility with technology ability a key differentiator initially	
Create a Strategic Vision	● Build a vision to guide change efforts	● Initial lack of strategic vision	● Initial focus reactionary rather than aligned Cross-disciplinary themes emerged
	● Envision and share a strategy for success	● Vision emerges as needs and goals identified through top-down communication	
		● Settling out process	
Communicate the Vision	● Communicate expectations ten times more than expected	● Bilateral communication including trickle-up of what was working	● Regular communications channels established both within and across specialties
	● Vary communication strategies	● Communicating uncertainty was reassuring	
	● Guiding coalition role models new behaviors		
Empower Others to Act on the Vision	● Remove or alter organizational obstacles	● New telemedicine, information management, and education roles for clinical trainees	● Co-creation of educational work designed to support rapidly identified clinical need
	● Support experimentation and rapid improvement cycles		
		● More self-regulated and self-directed learning	● More flipped classroom implementations
			● Medical students identify need for and implement educational sessions
		● Increase in learner ownership	
			● Allocate designated roles by best fit helps flatten hierarchy
Plan for and Generate Short-Term Wins	● Showcase short term, visible improvements	● Initially maintain moral through “non-losses”	● Continue educational mission without lapse
	● Publicly reward those who enable and support wins	● Level of engagement an important early guiding indicator of success	● Celebrate trial, error, and growth
			● Inclusive participation of teachers and learners within sessions
			● Realize advantages of online learning
Consolidate Improvements and Produce Still More Change	● Promote those who are effective change agents	● Identify what is working	● Increase in attendance at rounds and conferences
		● Challenge long-held assumptions about how clinical education should occur	● Use of chat, poll, and screenshare features
	● Energize the change by offering resources and supporting new projects		● Engage discussant, moderator, and reviewer roles to support integrated learning
Institutionalize New Approaches	● Sustain change by ensuring new approaches are understood by all	● Organizational change requires a predisposition to accepting change as a constant	● Accept iteration and ambiguity inherent in educational process
			● Trial new methods, learn from failures, and share what worked
	● Vocalize connections between new changes and organizational success		
		● Adaptation integrated into the clinician-educator role	● Recognize fallibility and humanize education

### Theme #1: responding in a climate of urgency

In this unique situation, respondents expressed a palpable sense of urgency. Faculty concerns involved their personal risk in participating in a frontline clinical environment, while medical students were concerned with being excluded from direct care, and both groups worried that the educational processes risked being rendered inadequate.

So we were asked to suddenly remove students from their normal [educational] life, [for example,] rotations - and try to suddenly make every activity virtual (T, #2)

[Me and my fellow Chief Residents] were asked to essentially turn all resident teaching into all virtual formats as soon as possible. We gave ourselves one week and for that time stopped all teaching, but along with changing to virtual we also were changing people’s schedules and dealing with a lot of furloughs. But we did it. We converted essentially everything to virtual, including morning report, noon conference, journal club, intern report, and Grand Rounds. It was crazy as we suddenly connected with 80 people or more over zoom. (L, #14)

Both teachers and learners had to define the tasks required to convert from in-person to interactive video-based clinical education almost overnight, both to ensure safety and mitigate the damage to educational processes. The most urgent tasks involved determining what could go online and how it could be adapted to the virtual environment. Feeling the urgency to keep the educational mission moving forward, some educators tried new pedagogic models they had only lightly implemented previously.

It was kind of all of a sudden we joined conferences over zoom. But then I switched to a new rotation and there was suddenly also a new expectation to actually deliver academic conferences. I worked with the chief resident, and it was just kind of like any former teaching conferences with some changes – you still prepared slides but you will deliver this differently. (T #22)

We've had to kind of reimagine how we were going to teach it. What we used to do is they would come in and hear my lecture. We'd go through cases and that would be the end of the day. What we did now is they were given material to preview the night before. And then I gave my lecture and then we kind of bent over backwards to make sure they had exposure to the topic before they heard their lecture, which is something we had kind of hoped to be able to do. But we were able to sort of institute it in this new model. (T, #30)

### Theme #2: guiding the shift with a differentially engaged leadership coalition

Both educators and learners soon realized that the cavalry was not coming to the rescue in terms of their educational goals. The guiding coalition initially consisted almost entirely of those already involved in frontline education. Not all those initially engaged for help were available when needed:

We did run into the issue of so much generational [difference] where some people were able to rather easily move into teaching over zoom... Others wanted nothing to do with it, but didn't even want to learn how to go about [the transition]... (T, #6)

Initially technological proficiency was an important determinant of who could design or lead the shift to interactive video-based clinical education. While minimal formal technical and educational support was offered, teachers and learners more adept with technology stepped in to support and lead educational initiatives in progress, serving as a technological scaffold to sustain efforts for all.

The urgent problem was people learning how to use those platforms. [Some were] pretty fast and some were already doing lectures via zoom. Actually, I [was giving] video lectures before the crisis. [But those who had never used zoom] had some difficulty, for instance, you know, like showing their slides… (T #2)

We did mostly case based teaching, with an expert discussant. I created the case but then a chief resident reviewed my slides before the conference. The chief resident was also on the zoom to help. (T, #22)

Teachers joined these efforts by curating educational experiences and resources for learners. The initial focus was on offering educational content, even if they were at times haphazard or misaligned.

We as faculty basically joined forces and came up with a list of topics to teach. It was kind of haphazard in a sense, but we tried to fill in the gaps and do our best. (T, #9)

### Theme #3: attempting to develop strategy and vision

Initially, the unexpected shift to virtual interactive learning lacked a strategic vision as to what was to be done and how, with adaptations occurring in a reactionary manner. Top-down communication from leadership was helpful in describing an overall situational awareness while also adapting to a growth mindset over performance.

So I learned to tell my students right at the start…to acknowledge, this [interactive video-based clinical education] is not going to be perfect. There are going to be problems, but let’s do it. (T, #18)

[Medical school leadership] in particular would help us with regards to keeping us informed [of the] very rapidly moving landscape as best they could with the idea that there was like, not much information that anybody had. At least that was consistent information. (T, #6)

This general information often trickled down to learners. Teachers were largely transparent with learners that plans were often being made and executed in tandem. Learners were encouraged to reach out to teachers with questions or concerns, which mitigated some anxiety surrounding the transition and whether they would meet learning objectives.

There was a lot of flexibility. We could reach out to faculty to discuss anything that would make it individually successful for us. So both our various preceptors for those different components as well as pure leadership. They made it clear that we could reach out with questions. (L, #13)

Over time, a vision for how interactive video-based clinical education would become integrated into the structure of training began to emerge. Both teachers and learners began to understand more about the why’s and how’s of delivering content online. The development was slow but at some points, organic.

As faculty started to figure [it] out, we began to see the reasoning behind why we are using things like online case like interactive platforms. We would then be able to plan for those sessions and dedicate enough time and effort. (L, #13)

Both teachers and learners recognized that while in-person interaction cannot be replicated, it can be augmented or adapted. This aligned with the broad goal of keeping educational initiatives afloat.

So while I think we transitioned rather quickly to virtual learning the first couple of conversations might not have been traditional knowledge or skills based content, but more of a conversation from a 30,000 feet view, as opposed to an ‘in the trenches’ view with regards to how the students were doing and what their experience was like. (T, #6)

### Theme #4: empowering a volunteer army through effective communication

Coalitions of individuals dedicated to the cause formed, sometimes as mandated by departmental leadership but other times organically. This included trainees being reassigned to support teaching or oversight of more junior trainees.

It is almost like we [clinical teachers] quickly began building a community. I could say I was one of the leaders building that community. We kept emailing each other all the time and learning from each other, asking “how do you do this” and “how do you do that”? (T, #18)

The residency program, the chiefs, and IT - it's kind of shocking how quickly it all came together. [The program] dedicated five full time internal medicine residents to it. They had others last block and they're switching them out. They loved the idea of the virtual resident really feeling like part of the team. (L, #10)

This extended to offering trainees assigned to work from home the opportunity to take on new and innovative roles to combat COVID-19. This distributed educational work developed in part to fill the gap aligned with current clinical need:

The residency program said, “we were thinking about maybe trying to create discharge helpers, maybe some of the residents at home could help prep discharges [and learn through that].” So it came out of a clinical need. (L, #10)

In some cases, trainees were engaged in the process of delivering education. This was driven in part by pure need to continue providing education, but also because trainees were sometimes more adept at using technology.

For an upcoming clinical reasoning session this Wednesday, [clinical educators] have had it that for all the small groups, the students themselves set up the zoom link.... [the students] are better at computers than the rest of us. (T, #1)

After time, stronger methods to engage multiple participants were identified. This functioned as a way to support faculty and trainees in co-creating some learning experience, as well as setting the new cultural norms of the educational experience.

[The students] decided…if you're on an outpatient block you're on deck to be called upon. The chief [residents] also designated a point person for me to ask on the inpatient teams. And someone monitored the chat. So giving people a head's up, like you should be aware you're going to be one of the participants today [during our learning session].” (T, #22)

### Theme #5: generating short-term non-losses

Continuation of the education mission was a key short term non-loss. Even taking different less-polished forms, the ongoing delivery of clinical skills education in and of itself was attained. That clinical experiences could in some way still exist was a source of pride for teachers.

[The students] actually could still continue to have some clinical interaction because we knew that the primary care doctors were doing a lot of virtual visits. (T, #5)

After some trial, error, and time, methods to engage more participants were identified. This in turn led to feelings that all were included in the new educational effort.

After repeat experiences we tried new things. We decided to have the entire class participate for everything on every question. It was really great because now everyone was contributing. (L, #12)

This was sometimes accompanied by the realization that online educational activities have certain advantages even compared to in-person activities.

The overall reaction [to interactive-video based clinical education] was very positive. And [the students] felt like they were really able to focus on their oral presentation skills and their clinical reasoning skills. Maybe even more so than what they do when they’re with a patient. (T, #1)

[The students] had more time than ever to prepare for things… they could really take the time to prepare [for] the flipped classroom… There was a lot more self-directed learning. (T, #11)

I've enjoyed this so much is because … I feel like I'm more able to bring in other resources to study (L, #21)

There was recognition that reducing in-person activities made it easier for some to join educational activities that they would have missed if required to be physically present.

Sometimes when [residents] were in the hospital before and everything was in person, it was impossible to even make it to conference. Now the teaching is all there and its 100%. I could just eat lunch and beautifully focus on teaching and learning. It makes it easier for me to participate. (L, #16)

### Theme #6: sustaining improvement and positive momentum

Initial efforts transitioned to an emphasis on continuous improvement of the use of the new educational technologies in clinical skills teaching.

Other hurdles have been around some of the physical diagnosis rounds. [The medical students] struggled with a little bit with the technology in terms of making sure that when we played murmurs that everybody can hear them. Or that when we play videos, everybody can actually see the video. And so that that has been another big issue we had to overcome. (L, #14)

Once features to increase engagement were identified, they were more consistently implemented. This was thought to drive both attendance and participation.

Virtual learning is actually more effective because we get greater attendance and participation. Especially the chat features. Learners are really willing and able to just quickly write something and contribute or show they don't understand. (T, #22)

The new educational work was codified into new learner and clinician roles in the virtual or hybrid environment, such as lead discussant, chat moderator, chart reviewer, or on-call respondent, integrating the learning experience more solidly for all.

The integrated nature of learning was better. We were having a case discussion, for instance, and several [students] were discussing the case. Another student was able to go through the chart. We then didn't have to make guesses about the clinical situation. Students were also able to answer details right away. (T, #2)

### Theme #7: considering plans for long-term change

As the uses for interactive video-based clinical education became recognized, early plans were developed to identify changes which might be sustainable over time. There was a realization that precious clinical time could be preserved through the maintenance of some virtual or asynchronous curricula.

If we can take these videos or video lectures and put them outside of the time that we are in the clerkship, just to add to the amount of time that we spent in the hospital, I think that will make it a much better experience. (L, #29)

As part of a desire to reach even more learners, some started recording morning reports, grand rounds, or teaching sessions. These sessions could now be made available to learners who could not attend the first time or those who wanted to revisit the session for additional learning.

[Teachers] are reaching more people and more people now go to report. We are now recording reports, which we have never done before. And people are watching them. I think the teaching is as good but we are getting more people. (L, #14)

There was also consideration of hybrid options during a post- COVID-19 future. Potential for increased use of flipped classroom with a reduction in in-person learning time was highlighted.

I wonder if there can be a transition from taking us out of the clerkships Wednesday mornings and I think Thursday afternoons and work more [in clinic] Wednesday mornings and then transitioning to a more video based learning. (L, #29)

With an eye to the future, participants recognized that prior methods of teaching and learning may not have been ideal. They were open to employing newer models which embed more interactivity with and involvement among learners.

When not in the OR, most teaching up to now have been one way, mostly lecture teaching. One of the things [teachers] are doing is to consider rolling out modules with upfront videos they can watch. We can then discuss and walk through aspects of the critical steps of a procedure. Then we can talk through permutations in an interactive fashion. (T, #9)

### Theme #8: adapting to adapting

Throughout this change process, teachers and learners continually adapted to the *process* of adaptation itself. The nature of the changes that could happen, as well as the pace of those changes, varied.

At the very beginning, when there was a lot of unknowns about how [learners] would be able to actually transition to a lot of these different components. I think that I would have said it would have been less effective. But now that we've seen a few iterations over the weeks, my guess is that yeah, it rose in its effectiveness. (L, #13)

Participants became used to this ambiguity, and accustomed to a rapid pace of change. While initially startling, this sometimes served as a forcing function to trial new teaching methods, learn from educational failures, and share tips with others.

A number of presenters have shared how they tried [something] and then [it] didn't work, and then I did this. And [teachers] kind of problem solved on their own and shared those tips, and [now] we're trying to send out tips to our faculty about things we've learned - like how you can do this successfully. (T, #5)

This adaptation process helped push teachers and learners outside of their comfort zone. This sometimes humanized education, with both teachers and learners realizing that neither group had all the answers.

The last piece is this idea of offering yourself, I think in a very kind of transparent or vulnerable kind of way. That, you know, we're not experts at this and, you know, if you have more questions, you really need to reach out to us because it's really hard for us to tell if you're getting it or not. (T, #7)

## Discussion

Change is difficult in the best of times. In describing a rapid organizational change to clinical skills learning we found important parallels, and differences, compared with more conventional, staged processes. Important differences revealed as part of our investigation were that the urgency for change was felt by all and made for a rapid alignment of stakeholders and openness to methods that had been in the wings all along. Also, rather than having a strategic vision developed and promoted centrally, we found that a consensus as to best methods emerged from the failures and successes of the early efforts. Thus our findings from the pandemic disruption to clinical skills education reinforce and supplement the notion that change at the level of a curriculum and its implementing organization follows consistent patterns, providing opportunities for beneficial intervention.

The results of our thematic analysis reveal many aspects of change that are consistent with the reports by others dealing with pivoting to online learning [26]. These include the use of remote synchronous and asynchronous educational activities [16, 23, 30], more interactive didactic or case-based teaching sessions [16, 31, 32], replacing in-person clinical rotations with clinical telemedicine experiences or virtual teaching rounds [30, 33–36], clinical service reconfigurations [30, 37], unexpected learning experiences [38], and the need for adaptability [6, 15, 17, 30, 38–41]. Aligning with prior work, we note that some face-to-face activities cannot be effectively replicated in the virtual environment, yet we also expect some changes to persist past the pandemic [30, 39–42].

We also discovered several aspects of changed behavior that have been less widely reported and thus offer novel contributions to the literature, including collaborative efforts between faculty and trainees to support clinical and didactic education efforts, and the importance of bidirectional communication during this transition [32, 43]. There was also a noted differential in technological adeptness, likely due in part to prior personal experience with technology and in part due to generational differences, that translated to different levels of influence on the subsequent changes. We also note that the reduction of in-person activities was in some ways a welcome change, and offered even more opportunity for some to join synchronous learning. As reinforced by other recent findings [26, 44], participants desired to maintain some reduction of in-person synchronous learning time post-COVID, and recognized that traditional methods of teaching and learning may no longer be ideal for all educational experiences.

Our findings can be considered in the context of one or more recognized theories of crisis management and conventional organizational change. As we coded the reports of our learners and educators, we felt that the Kotter framework for planned change management made for a strong conceptual alignment with the constructed themes, and in particular the role of "urgency" in bringing about change[45]. Once the urgency was understood, the process became about channeling the urgency so as to not only mitigate the disruption but also to explore new affordances. It might have been expected that because the pandemic forced immediate and unanticipated change, more explicit crisis management frameworks would have been more appropriate [46–49], considering important elements such as type of crisis, threat level, degree of control, time pressure, and number of response options. In a crisis model, response options to approach the problem typically decrease as the crisis continues [46]. However, our participants found they sometimes had more choice moving forward, by for example engaging trainees in needed clinical tasks while working from home or including trainees in the process of delivering education. While our participants were under time pressure to make decisions quickly and without knowing all the facts – as would be expected [9, 46]—once the initial push to immediately transition to interactive video-based clinical education was complete, they were able to thoughtfully consider what elements worked and what did not. In a crisis model, resources are provided abundantly to solve the problem [49]. However, our participants were encouraged to identify and use existing human capital and technological resources rather than being offered financing to, for example, outsource transition efforts to a for profit education management company.

Moreover, while crisis management often focuses on minimizing damage and ensuring organizational survival [50], we found evidence that our participants worked collaboratively towards a positive outcome by continually addressing challenges and learning from them. There was no sense that the educational mission could or would fail. Ultimately, if participants had utilized a crisis management model, the overarching emphasis is on reaction and restoring the status quo as quickly as possible.[46–51]. This might have lessened participants capacity to use the pandemic crisis as an opportunity to deliberately consider which changes could be beneficial in the medium term and should be maintained post-COVID, the latter perspective being more consistent with change management.

For all the near-heroic efforts put in by educators and trainees to keep the ship afloat during this storm, the most interesting outcome, yet to be determined, is the extent to which Kotter’s markers of success, sustained and long-term institutional change, will persist in the clinical education of medical students. The clear *healthcare* response at the time was to quickly adopt a command and control structure. This did not seem to be the response within education which continued a distributed model. In this regard, our data suggest both teachers and learners have had their eyes opened to new and variable ways of doing things all along. While many aspects of clinical education will undoubtedly revert to past methods, educators and trainees alike have come to a set of new realizations about optimizing the blend between physical and virtual activities and have explored new roles for learners.

### Limitations and future research directions

This work has a number of limitations. First, we conducted our interviews at a single site early in the COVID-19 pandemic, thus our participant experiences are not reproducible and may not represent the experiences of other institutions. Second, because we purposefully interviewed teachers and learners across the educational spectrum, we captured broad experiences rather than the intensive reflections of any particular group. Third, our interviews were conducted with quickly recruitable volunteer participants, and thus may not be representative of all teachers and learners throughout our institution. While many of our participants are in leadership roles, we chose not to approach the broader leadership of the medical school such as the Deans of the undergraduate or postgraduate programs in order to focus on front-line educators and learners. Thus, while we chose to focus on trainees and clinical educators, we are limited in our understanding of how attempts to actively manage change were navigated at the highest levels of institutional leadership. In the future, the degree of education leaders buy-in to the changes made may determine the extent of their long-lasting effect. However, more recent work suggests that healthcare C-suite leadership response to the pandemic may also align with Kotter’s change management model; thus our findings are likely complementary [45, 52]. We also note that interviews were conducted during the early stages of the pandemic while rapid change was ongoing, thus outcomes and lessons learned likely continued to adapt. We also acknowledge that the Kotter framework informs but does not completely explicate our themes. Future research should investigate the role that educators and students have in effecting change, as our work points to them having more power than perhaps previously understood. Additional work might address whether there was a change in the quality of clinical education as a result of the pandemic. Finally, studies could test the necessity of all eight Kotter stages in situations with natural urgency and a more distributed decision making model, which might align with a more contemporary clinical care structure.

In summary, our findings suggest that crisis-induced change and planned change show significant homology in the overall process but differ in emphasis, with some aspects being paradoxically easier during the crisis. The story remains to be told as to which changes will be continued but clearly a unique set of circumstances created a context where change became possible.

## Conclusion

We used a phenomenologic paradigm eventually informed by a change management framework to understand how an academic medical center navigated an extremely rapid and unexpected transition to a new method of clinical education. Our findings extend prior literature by offering an understanding of how standard principles of change management relate to crisis adaptation, changing the order and importance of some of the stages of rational change. Disruptive change in time of crisis can lead to opportunities when systems adapt effectively.

## Supplementary Information

**Additional file 1.**

## Data Availability

The datasets generated and/or analyzed during the current study are not publicly available because we did not seek permission from our participants or Institutional Review Board to make the data publicly available, but are available from the corresponding author on reasonable request.
