# Supplementary material for: Educational adaptation to clinical training during the COVID-19 pandemic: a process analysis
Source: BMC Med Educ. 2022 Mar 23;22:200. doi: 10.1186/s12909-022-03237-6 (PMC8942055; doi:10.1186/s12909-022-03237-6)
Supplement: Supplementary file 1 — Additional file 1. [file 12909_2022_3237_MOESM1_ESM.docx]

**Appendix: Interview Guide**

- **Over the last several weeks of the COVID-19 crisis, please describe the process by which you first transitioned to video-based teaching and learning.**
  - How were you asked? What was the proposed timeline provided? (1 min)
  - What specifically was the task requested of you? (1 min).
  - What specifically was the new education curriculum incorporating video learning (1 min).
- **Please describe your experience with your first 2 experiences using video-based teaching and learning. What was it like? How did it go?** (~2 min)
- **After multiple video-based teaching and learning experiences (your new steady state)**
  - What hurdles, difficulties, or unexpected problems were encountered? (1 min)
  - How were they solved, & how did you adapt? Please give 1-3 examples. (~2 min)
- **Assuming your prior teaching/learning environment (before video transition) is a baseline of “100% effective”, please estimate the level of educational value compared to baseline that you believe is achieved by the implementation of the new video-based teaching and learning.**
  - Please list the percentage compared to baseline for your primary video-based teaching and learning (~10 sec)
  - Please describe your answer and how you made this estimation (~2 min) “e.g…because…”
- **From your viewpoint, did the new educational environment using video learning change behaviors in the learner?** Describe (2 min)
- **From your viewpoint, did the new educational environment using video learning afford the learner any new benefits?** Describe (2 min)
- **Finally, what, if any, of the video based efforts do you think will continue to be utilized for your educational efforts, once the COVID-19 crisis has resolved**? (1 min)
